# Evaluating the Agreement of Risk Categorization for Fetal Down Syndrome Screening between Ultrasound-Based Gestational Age and Menstrual-Based Gestational Age by Maternal Serum Markers

**DOI:** 10.1155/2018/9687042

**Published:** 2018-03-08

**Authors:** Pakorn Chaksuwat, Supatra Sirichotiyakul, Suchaya Luewan, Theera Tongsong

**Affiliations:** Department of Obstetrics and Gynecology, Faculty of Medicine, Chiang Mai University, Chiang Mai, Thailand

## Abstract

**Objective:**

To evaluate the agreement of risk categorization for Down syndrome screening between ultrasound scan-based gestational age (GA) and last menstrual period-based gestational age in both first and second trimesters by maternal serum markers.

**Methods:**

Data comprising 4,055 and 4,016 cases of first and second trimester screening were used. The maternal serum markers were analyzed using the ultrasound-based GA and menstrual age. The subjects whose menstrual age and ultrasound-based GA fell in different trimesters were excluded because the risk could not be calculated due to the different serum markers used in each trimester. The agreement of risk categorization for fetal Down syndrome was evaluated.

**Results:**

The agreement of Down syndrome screening in the first and the second trimesters were 92.7% and 89%, respectively. The study found a good agreement of risk categorization by Kappa index, which was 0.615 for the overall screening. The menstrual age had a slight decrease in the detection rate and a lower false-positive rate.

**Conclusion:**

Menstrual age is acceptable in cases of accurate last menstrual period. However, in places where ultrasonography is not readily available, gestational age estimation by menstrual age along with clinical examination that corresponds to the gestational age can be reliable.

## 1. Introduction

Currently, screening for Down syndrome should be available to all women who present themselves for prenatal care before 20 weeks of gestation regardless of maternal age [1]. Screening tests using maternal serum markers are the effective and acceptable test for Down syndrome in the general population. The screening test can be divided into two periods of gestational age: the first and second trimester screenings. For the first trimester screening, the two serum markers used were free beta-human chorionic gonadotropin (free beta-hCG) and pregnancy-associated plasma protein A (PAPP-A). For the second trimester screening, triple or quadruple tests can be used. The triple test consisted of alpha-fetoprotein (AFP), free beta-hCG, and unconjugated estriol (uE3), with the addition of inhibin A for the quadruple test. The detection rate of Down syndrome using serum markers in the first trimester and the triple test in the second trimester are 67% and 69%, respectively, at a 5% false-positive rate [2, 3].

The correct gestational age is an essential factor that ensures accurate screening using maternal serum markers in both first and second trimester because the multiple of median (MoM) of each marker depends on gestational age. Incorrect gestational age may result in a false-positive or false-negative test. A false-positive test leads to unnecessary invasive prenatal diagnosis and increased risk of miscarriage from the procedures.

The gestational age can be estimated using ultrasound scan-based gestational age (US-based GA) and last menstrual period-based gestational age (LMP-based GA). In the general practice of Down syndrome screening, maternal serum markers are usually calculated and evaluated by US-based GA, which is the standard method used worldwide. However, the disadvantage of using US-based GA is the need for an ultrasound machine and a trained ultrasonographer, which are not available in all areas and constitute a rather huge burden on healthcare system in our country, especially in the rural areas.

The objective of this study is to evaluate the agreement of risk categorization for fetal Down syndrome between the US-based GA and LMP-based GA in both the first and second trimester screening tests by maternal serum markers.

## 2. Materials and Methods

This study was ethically approved by the institute review boards (Chiang Mai University, Thailand). The data were retrieved from the Down syndrome screening database of the maternal-fetal medicine unit from January 2011 to December 2014. All pregnant women underwent an ultrasound to estimate the gestational age using crown-rump length (CRL) and biparietal diameter (BPD) for the first and second trimesters, respectively. The exclusion criteria included multifetal pregnancy, fetal anomaly, underlying diseases that may interfere with the maternal serum marker value (such as chronic kidney disease with or without the need for hemodialysis), incomplete data (such as no LMP recorded, no fetal outcome), and LMP-based GA and US-based GA that were not in the same trimester.

Free beta-hCG and PAPP-A were used for the first trimester screening test at the gestational age of 9^0/7^–13^6/7^ weeks, while the triple test (AFP, free beta-hCG, and uE3) was used for the second trimester screening test at the gestational age of 14^0/7^–20^5/7^ weeks. All pregnant women were categorized into high risk (1 : ≤250) and low risk (1 : >250) in both the first and second trimester screening tests.

The data used in this study were based on our prospective database which had been developed for the program of fetal Down syndrome screening under the National Research University Project of Thailand. All newborns in this program were prospectively assessed by pediatricians in the project team. Cytogenetic study was done only when chromosomal abnormality was suspected by the pediatricians. The diagnosis of Down syndrome was based on chromosome studies either by antenatal or postnatal studies, whereas the diagnosis of non-Down syndrome was based on chromosome studies or the conclusion by the pediatricians in cases of no chromosome study results.

The maternal serum markers were analyzed using the US-based GA and LMP-based GA by PerkinElmer LifeCycle software version 3.2 in both first and second trimester screenings. Statistical analyses were performed with SPSS for Windows statistical package version 21.0 (IBM Corp., Armonk, NY). The intraclass correlation coefficient statistics and Cronbach's alpha were calculated to assess the agreement between the two methods of determining gestational age, the US-based GA and LMP-based GA. Cohen's Kappa statistics was used to test the agreement of risk categorization between the US-based GA and LMP-based GA with *p* < 0.05 considered statistically significant.

## 3. Results

**Figure 1:**
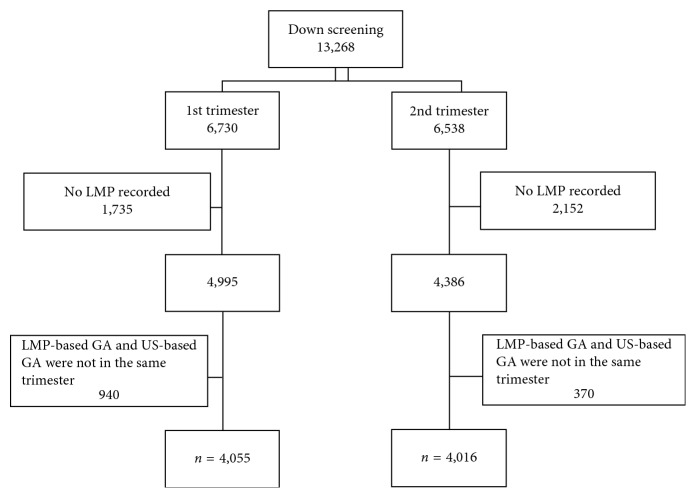
The number of Down syndrome screening in the first and second trimester.

During the study period, 13,268 pregnant women were enrolled into the screening tests. LMP was not available in 3,887 cases (1,735 and 2,152 cases for the first and second trimesters, resp.). Also, 1,310 cases were excluded because the LMP-based GA and US-based GA were not in the same trimester (940 and 370 cases for the first and second trimesters, resp.). In total, 8,071 cases were available for analysis as shown in Figure 1.

The demographic data of the 8,071 cases enrolled into the study are categorized into first and second trimester screenings as shown in Table 1. The population studied did not include residents of the capital city. The mean ± SD of the maternal age was 29.2 ± 5.6 years; the range was 13.7–45.8 years. The mean ± SD of the maternal weight was 55.5 ± 10.1 kg; the range was 30.5–138.0 kg. The mean ± SD of the gestational ages at sampling for the LMP-based GA and US-based GA in the first trimester screening group were 87.2 ± 6.3 days (70–97 days) and 87.1 ± 5.3 days (70–98 days), respectively. In the second trimester screening group, they were 112.9 ± 9.2 days (98–146 days) and 111.5 ± 7.2 days (98–142 days), respectively.

**Figure 2:**
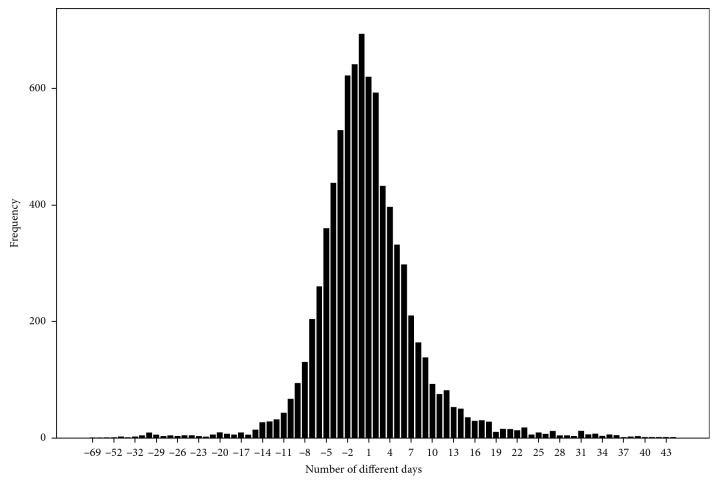
The distribution of the number of different days between LMP-based GA and US-based GA in gestational age estimation.

The difference between the gestational ages based on LMP and US was analyzed and categorized into seven groups, as shown in Table 2. We found that there was no difference in 8.6 % of the gestational ages, while the difference was within ±7 days in 82.1% and within ±14 days in 95.3% of the gestational ages. The distribution of the gestational age differences is shown in Figure 2. The agreement between the two methods in determining the gestational age was analyzed. The intraclass correlation coefficient (ICC) of the gestational days between the two methods (US-based method and LMP-based method) was 0.936 (95% CI: 0.933–0.939, *p* < 0.0001; Cronbach's alpha: 0.936). Therefore, the agreement between the two methods in determining gestational age was excellent.

There were 1,310 women (14%) whose LMP-based GA and US-based GA were not in the same trimesters. They were analyzed and categorized as shown in Table 3. We found that 50% of this population had GA discrepancy of more than 2 weeks.  Table 4 shows the overall result of Down syndrome screening and the frequency of (high/low) risk category of the screening. The number of women in the high-risk group using the US-based GA and LMP-based GA was 340 (8.4%) and 468 (11.5%) in first trimester screening and 587 (14.6%) and 825 (20.5%) in the second trimester screening, respectively. We found 20 Down syndrome fetuses from the total of 8,071 fetuses. The sensitivity and specificity of the US-based GA were 75.0% (95% CI: 56.0%–94.0%) and 88.7% (95% CI: 88.0%–89.4%), respectively, while the sensitivity and specificity of the LMP-based GA were 70.0% (95% CI: 49.9%–90.1%) and 84.1% (95% CI: 83.3%–84.9%), respectively. The positive predictive value (PPV) of the screening performances based on the US-based GA and LMP-based GA was 1.6% (95% CI: 0.8%–2.4%) and 1.1% (95% CI: 0.5%–1.6%), respectively. The negative predictive value (NPV) was 99.9% (95% CI: 99.7%–100.0%) in both groups. The odds of being affected by a positive result (OAPR) of the screening performances based on the US-based GA and LMP-based GA were 1 : 63 (95% CI 1 : 100–1 : 37) and 1 : 91 (95% CI 1 : 143–1 : 55), respectively.

The agreement of risk categorization between the US-based and LMP-based screenings is shown in Table 5. The percentage of agreement of the overall Down syndrome screening in the first, second, and both trimesters was 92.7%, 89%, and 90.9%, respectively. The agreement of risk categorization was analyzed, and we found a good agreement of risk categorization between the US-based GA and LMP-based GA by Kappa index, which was 0.615 for the overall screening and 0.592 and 0.622 for the first and second trimesters, respectively.

## 4. Discussion

Our study showed that the agreement of gestational age estimation between the LMP-based GA and US-based GA was excellent in the population studied. We found that the mean LMP-based GA in the second trimester group was slightly greater than that of the US-based GA (1.37 ± 7.9 days). This corresponds to previous studies which showed that the LMP-based GA was 2.3 days greater [1, 2].

In a certain population, the gestational age discrepancy between the LMP-based GA and US-based GA in more than 80% of women was less than 7 days. This is consistent with previous studies [3, 4]. In the first trimester group, a gestational age discrepancy of less than 7 days was found in 86.7% of the women. For the second-trimester group, a gestational age discrepancy of less than 14 days was found in 92.8% of the women. According to the guidelines for ultrasound-based redating, these populations would not be redated [5]. This high rate of gestational age accordance is probably due to the practice of the recorder who chose to record LMP in women who were certain of their LMP, rather than those who had irregular menstrual periods prior to pregnancy and those who were uncertain about their LMP. As a result, in a pregnant woman with certain LMP, the LMP-based GA was rather reliable. Therefore, using ultrasound to confirm gestational age in this group of women might be unnecessary. However, if the LMP-based GA did not correspond to gestational age by clinical examinations, ultrasound may play a vital role in confirming gestational age.

The result of this study shows that the detection rate of the US-based GA is slightly better than the LMP-based GA. The LMP-based GA had a slight decrease in the detection rate and a higher false-positive rate. In this study, the screening positive rate (SPR) of Down syndrome screening was about 11% when the US-based GA was used, which is a little bit higher compared to 6–8% from previous reports [6, 7]. The difference might be due to the fact that the software that we used came with the reference value of the Caucasian population. Previous studies have shown that in the Asian population, even when the confounder such as maternal weight was corrected, the SPR is still higher [6, 8, 9]. If this software is used with the Asian population, it may increase the number of invasive prenatal diagnosis performed. If we use the Asian reference, the SPR in this study may be close to that of other studies [8].

The objective of this study is to evaluate the agreement of risk categorization between the LMP-based GA and US-based GA. The screening using the LMP-based GA led to higher SPR but slightly lower detection rate, which corresponded to previous studies [1, 2, 10]. However, the agreement of categorization of Down syndrome screening was good. Thus, we can infer that in pregnant women whose last menstrual period was certain and whose clinical examination was consistent with LMP-based GA, the LMP-based GA is an acceptable method to be used for Down syndrome screening when ultrasonography is not available. When the Down syndrome screening falls into high-risk category, gestational age reevaluation may be performed to reduce SPR. However, the drawback of this approach is that pregnant women and their family may experience anxiety if the initial result is high risk and the latter result after US-based GA adjustment shows low risk.

The limitation of this study is that we had to exclude the group of the population whose the LMP-based GA fell into a different trimester from the US-based GA because we could not calculate the risk result in those cases. However, in the excluded population, almost 50% of them had gestational age discrepancy of more than 2 weeks. In general, this group of population can be detected by clinicians since clinical examination would show the discrepancy and they would be further investigated by ultrasonography.

## 5. Conclusion

The reliability of the LMP-based GA in our population was generally acceptable. For Down syndrome screening by maternal serum markers, the US-based GA was slightly better than the LMP-based GA. The LMP-based GA was acceptable in cases of accurate LMP, with a slight decrease in the detection rate and a higher false-positive rate. However, in places where ultrasonography is not readily available, gestational age estimation by LMP can be done with caution. When clinical estimation is not correlated with the LMP-based GA, the patients should be sent for further investigation by ultrasonography.

## Figures and Tables

**Table 1:** Demographic data.

	Mean ± SD	Range
Maternal age (year)		
1st trimester screening	29.05 ± 5.6	
2nd trimester screening	29.3 ± 5.7	
Total	29.2 ± 5.6	13.7–45.8
Maternal weight (kilograms)		
1st trimester screening	54.9 ± 10.0	
2nd trimester screening	56.1 ± 1.0	
Total	55.5 ± 10.1	30.5–138.0
Gestational age (days)		
1st trimester screening		
LMP^∗^ dating	87.2 ± 6.3	70–97
US dating	87.1 ± 5.3	70–98
2nd trimester screening		
LMP dating	112.9 ± 9.2	98–146
US dating	111.5 ± 7.2	98–142

^∗^LMP, last menstrual period; US, ultrasound.

**Table 2:** The number of different days between LMP-based GA and US-based GA in gestational age estimation.

Mean different days	First trimester		Second trimester		Total	
	*N*	%	*N*	%	*N*	%
0	376	9.3	318	7.9	694	8.6
1–3	1837	45.3	1600	39.8	3437	42.6
4–7	1302	32.1	1194	29.7	2496	30.9
8–14	451	11.1	615	15.3	1066	13.2
15–21	50	1.2	163	4.1	213	2.6
22–28	18	0.4	70	1.7	88	1.1
>28	21	0.5	56	1.4	77	1.0
Total	4055	100.0	4016	100.0	8071	100.0

**Table 3:** The number of different days between LMP-based GA and US-based GA in gestational age estimation for those excluded due to trimester discrepancy.

Mean different days	First trimester		Second trimester		Total	
	*N*	%	*N*	%	*N*	%
1–3	67	7.1	20	5.4	87	6.6
4–7	168	17.9	69	18.6	237	18
8–14	251	26.7	83	22.4	334	25.4
15–21	154	16.4	37	10	191	14.5
22–28	106	11.3	44	11.9	150	11.4
>28	194	20.6	117	31.6	311	23.7
Total	940	100	370	100	1310	100

**Table 4:** Down syndrome screening results and frequency of high/low-risk categorization according to the type of screening.

	High risk, *n* (%)	Low risk, *n* (%)
US-based screening		
(i) Total	927 (11.5%)	7144 (88.5%)
(a) First trimester	340 (8.4%)	3715 (91.6%)
(b) Second trimester	587 (14.6%)	3429 (85.4%)
(ii) Down syndrome detected	15/927	5/7144
LMP-based screening		
(i) Total	1293 (16.0%)	6778 (84.0%)
(a) First trimester	468 (11.5%)	3587 (88.5%)
(b) Second trimester	825 (20.5%)	3191 (79.5%)
(ii) Down syndrome detected	14/1293	6/6778

**Table 5:** Agreement of risk categorization between US-based and LMP-based screening.

US-based screening		LMP-based screening		Total	Kappa index
		High risk	Low risk		
First trimester	High risk	**255 (6.3%)**	85 (2.1%)	340 (8.4%)	0.592
	Low risk	213 (5.3%)	**3502 (86.4%)**	3715 (91.6%)	
	Total	468 (11.5%)	3587 (88.5%)	4055 (100.0%)	
Second trimester	High risk	**485 (12.1%)**	102 (2.5%)	587 (14.6%)	0.622
	Low risk	340 (8.5%)	**3089 (76.9%)**	3249 (85.4%)	
	Total	825 (20.5%)	3191 (79.5%)	4016 (100.0%)	
First and second trimester	High risk	**740 (9.2%)**	187 (2.3%)	927 (11.5%)	0.615
	Low risk	553 (6.9%)	**6591 (81.7%)**	7144 (88.5%)	
	Total	1293 (16.0%)	6778 (84.0%)	8071 (100.0%)	

Bold indicates the number (%) of agreement of the two types of screening.
